# Impact of a high Edinburgh Postnatal Depression Scale score on obstetric and perinatal outcomes

**DOI:** 10.1038/srep33544

**Published:** 2016-09-23

**Authors:** Pathmila Navaratne, Xin Y Foo, Sailesh Kumar

**Affiliations:** 1 Mater Research Institute - University of Queensland, Level 3 Aubigny Place, Raymond Terrace, South Brisbane, Queensland, QLD 4101, Australia; 2 School of Medicine, The University of Queensland, Brisbane, Australia

## Abstract

The aim of this retrospective study was to characterise intrapartum and neonatal outcomes in women with an antenatally recorded Edinburgh Postnatal Depression Score (EPDS) ≤ 9 compared with women with a score of ≥12 at a major Australian tertiary maternity hospital. Women with scores ≥12 are at particularly high risk of major depressive symptomatology. There were 20512 (78.6%) women with a score ≤ 9 and 2708 (10.4%) had a score ≥ 12. Category 1 caesarean sections where there was immediate threat to life (maternal or fetal) were more common in women with EPDS scores ≥12 (5.2% vs. 4.3%, OR 1.24 95% CI 1.03–1.49, p = 0.024). Pre-term birth (<37 weeks) was also more common (11.7% vs. 8.6%, OR 1.38 95% CI 1.21–1.57, p < 0.001). Women with high scores had higher rates of babies with birth weights <5^th^ centile (6.2% vs. 4.4%, p < 0.001). Apgar score < 7 at 5 minutes were more frequent in the high EPDS group (3.1% vs. 2%, OR 1.52 95% CI 1.18–1.93, p < 0.001). Resuscitation at birth (34.4% vs. 30.6%, p < 0.001) and neonatal death (0.48% vs. 0.13%, OR 2.52 95% CI 1.2–5.0, p < 0.001) were higher in babies of these women. These results suggest poorer intrapartum and neonatal outcomes for women with high EPDS scores.

The most common perinatal mental health disorders are depression and anxiety, however other conditions such as eating, personality and bipolar disorders, schizophrenia and psychotic illnesses are also not uncommon during pregnancy and the post-partum period^1^. The prevalence of major or minor depressive episodes during pregnancy is in the region of 6.5–12.9%, with almost 19% of women having a major episode in the first three months post-partum^2^. Risk factors for depression during pregnancy include low socioeconomic status, smoking, young age, unintended pregnancy, low income, lack of social support, life stress and domestic violence. There is evidence that obstetric and perinatal outcomes are worse in women^3^ with mental health problems with increased rates of complications such as preterm birth, low birth weight, neonatal intensive care unit (NICU) admission as well as increased risks of operative deliveries^4^,^5^,^6^,^7^. However not all studies have replicated these findings suggesting that the link between depression and adverse obstetric and perinatal outcomes may not be entirely straightforward^8^,^9^.

There are several screening tools that have been validated for use during pregnancy and the postpartum period to assist with identifying women, either at risk of, or suffering from perinatal depression^10^. However despite this, screening for depression is controversial because it has not been consistently shown to be of benefit and this uncertainty is reflected in various national guidelines and policies^11^.

The 10-item Edinburgh Postnatal Depression Scale (EPDS) is a widely used screening tool for depressive symptomatology and has been validated, both as an antenatal and postpartum screen for minor or major depression^12^. Although the EPDS is a screening tool for postpartum depression and not necessarily diagnostic for antenatal depression, it is generally accepted that high scores (≥12) are strongly indicative of major or minor depressive symptomatology. The EPDS is easy to administer and effective in its function, with high sensitivity and specificity. In addition, there is also evidence that the EPDS also measures symptoms of anxiety^13^,^14^ and therefore could serve as a proxy for both depression and anxiety. The purpose of this study was thus to correlate the results of the EPDS with intrapartum and neonatal outcomes at a major tertiary centre in Australia.

## Materials and Methods

This was a retrospective observational study of women birthing under the public health system at the Mater Mothers’ Hospital in Brisbane, Australia between October 2007 and December 2013. Only women with non-anomalous pregnancies **without** documented pre-existing mental health issues were included in the study. The Mater Mothers’ Hospital is one of the largest tertiary obstetric hospitals in Australia with almost 10000 births per year. Maternal demographic, intra-partum and neonatal outcome data were collected from the hospital’s electronic maternity database. Gestational age was calculated from either the last menstrual period or earliest ultrasound examination. Maternal body mass index (BMI) was calculated based on measurement of height and weight recorded at the booking visit.

Demographic information collected included maternal age, body mass index (BMI), ethnicity, and country of birth, parity and maternal medical conditions (hypertension, gestational diabetes, thyroid disease). Intra-partum outcomes included mode of delivery, duration of labour and length of stay in hospital. Caesarean sections were categorised as planned or emergency including the degree of urgency. Classification of the degree of urgency of caesarean section is generally based on one of four categories^15^: Category 1 - immediate threat to life (maternal or fetal); Category 2 - maternal or fetal compromise that is not immediately life-threatening; Category 3 - needing early delivery but no maternal or fetal compromise; or Category 4 - delivery at the convenience of the patient or obstetric team. Professional bodies such as the American College of Obstetrics and Gynaecology, National Institute of Clinical Excellence in the United Kingdom and the Royal Australian and New Zealand College of Obstetricians and Gynaecologists all have broadly similar recommendations in that the decision to delivery interval for Category 1 caesarean sections should be no longer than 30 minutes. Perinatal outcome data included gestation at delivery, birth weight, Apgar scores < 7 at 5 min, umbilical artery pH < 7.2 and/or lactate > 4 mmol/L, need for resuscitation at birth, respiratory distress, infection, admission to the neonatal intensive care unit (NICU) and neonatal death.

All women completed the EPDS questionnaire at their booking visit regardless of gestation. For the majority of women this took place at <20 weeks gestation. Each of the 10 questions was scored by the patient on a 4-point scale from 0–3 with a possible total score ranging from 0 to 30. We stratified our study cohort into two groups: low score (≤9) and high score (≥12). This categorisation has been shown to be predictive of the severity of depression with scores ≥12 indicating particularly high risk of major depressive symptomatology^16^.

Statistical analysis for this study was performed using the R and the R commander program (R Foundation for Statistical Computing, Vienna, Austria). Categorical variables were compared using a Chi-squared test. Continuous normally distributed variables were compared using a two sample t test and non-normally distributed variables were compared using a Wilcoxon rank-sum test. Multivariate logistic regression, adjusted for maternal age, BMI and parity was used to examine the effect of EPDS scores on intra-partum and neonatal outcomes. The level of significance was set at 0.05. Summary statistics are reported as number (percentage) and median (inter-quartile range) as appropriate.

Ethical and governance approvals were granted by the Mater Human Research Ethics Committee and Research Governance Office respectively (Ref no: HREC/14/MHS/63).

## Results

Over the study period there were 31071 non anomalous, public births at the Mater Mothers’ Hospital. We excluded 4961 women who did not have a recorded EPDS score leaving a final study cohort of 26110. There were 2708 women (10.4%) with an EPDS score ≥ 12, 2890 women (11.1%) with a score of 9–11 and 20512 women (78.5%) with a score of <9. The majority of women (88%) had the EPDS recorded at <20 weeks gestation.

### Maternal demographics

Women with EPDS scores ≥ 12 were younger, born outside Australia and be significantly more obese (BMI > 40 kg/m^2^) compared to women with a score of <9. There was also a higher prevalence of indigenous women in this group. Maternal medical diseases (hypertension and thyroid disease) were more common in women with high EPDS scores. These women also had a longer hospital stay Table 1.

### Intrapartum outcomes

There was no difference in the rates of normal vaginal deliveries, instrumental births or planned caesarean sections in women with high EPDS scores compared with women with low scores. Emergency caesarean section rates were however higher in women with scores >12 compared to those with scores <9 (19% vs. 17.5%, OR 1.16 95% CI 1.04–1.29, p = 0.006). Category 1 caesarean sections where there was immediate threat to life (maternal or fetal) were more common in women with EPDS scores ≥ 12 (5.2% vs. 4.3%, OR 1.24 95% CI 1.03–1.49, p = 0.024) Table 2.

### Neonatal outcomes

Gestation at delivery was similar in both groups although there were greater rates of pre-term deliveries (<37 weeks) in women with EPDS scores ≥ 12 compared to those with scores <9 (11.7% vs. 8.6%, OR 1.38 95% CI 1.21–1.57, p < 0.001). Overall, women with high scores had babies with lower median birth weights (3330 g, IQR 2950–3696.3 g vs. 3420 g, IQR 3066–3765 g, p < 0.001), higher rates of babies with birth weights <10^th^ centile (12.6% vs. 9.2%, p < 0.001) and <5^th^ centile (6.2% vs. 4.4%, p < 0.001). Neonates with an Apgar score <7 at 5 minutes were also more frequent in the high EPDS group (3.1% vs. 2%, OR 1.52 95% CI 1.18–1.93, p < 0.001). Rates of NICU admission (4.7% vs. 3.7%), need for resuscitation at birth (34.4% vs. 30.6%, p < 0.001) and neonatal death (0.48% vs. 0.13%, OR 2.52 95% CI 1.2–5.0, p < 0.001) were all greater in women with high EPDS scores Table 3.

## Discussion

The results from this study demonstrate that women with high EPDS scores have higher rates of emergency caesarean section particularly for concerns of fetal wellbeing. Our results also indicate that these women were significantly obese (BMI > 40 kg/m^2^), younger, of indigenous origin or more likely to have been born outside Australia. We also found a higher prevalence of maternal medical diseases (hypertension and thyroid disease) in women with high EPDS scores. We also found an association with low birth weight and pre-term birth and high maternal EPDS (≥12) scores. More worryingly however we demonstrate a higher prevalence of poorer newborn condition at birth, higher rates of NICU admission and neonatal death. To our knowledge some of these findings have not been previously reported.

Perinatal mental health disorders are important clinically, as up to 15–20% of women may be affected and this can impact maternal, fetal and neonatal outcomes. There is also a higher prevalence of depression and anxiety in women from low and middle income countries compared to those from high-income countries^17^. It is frequently unrecognized in pregnancy because depressive symptoms such as insomnia, poor appetite and decreased libido may be attributed to the normal physiological changes that occur during pregnancy and the puerperium. Furthermore, many women may be reluctant to report changes in their mood. Perinatal depression is often associated with poorer outcomes for mother–infant bonding and subsequent relationship and marital quality. There is good evidence that women with chronic psychotic conditions particularly schizophrenia are more likely to have adverse obstetric and neonatal outcomes including congenital malformations, preterm birth, fetal growth restriction, stillbirth, neonatal and sudden infant deaths. Some of these complications may however be related to the medication these women receive^18^,^19^,^20^,^21^.

In some other studies antenatal depression has not been shown (in contrast to our study) to be associated with hypertensive disorders, low Apgar scores or admission to NICU^22^. Although the risk of low birth weight and preterm birth may be increased in women with depression, the magnitude of this effect appears to be influenced to some degree by socioeconomic status and country of origin, with an increased risk seen in low and middle income countries (LMIC)^23^.

Some researchers have attempted to correlate fetal and neonatal outcomes with the use of antidepressants in pregnancy rather than with an actual diagnosis of depression. Although this is usually because of the availability of data of prescribing trends, it is nevertheless difficult to disentangle the effect of antidepressants, life style confounders, and depression^24^,^25^.

Depression and anxiety are both associated with increased concentrations of stress hormones. In particular, norepinephrine can cause uterine artery constriction^5^ which may reduce both antenatal and intrapartum utero-placental perfusion. This could potentially impair fetal growth and increase the risk of intrapartum fetal compromise precipitated by uterine contractions in labour. Another hormone released during stress is corticotrophin-releasing hormone (CRH) with higher concentrations found in women with depression and anxiety disorders. As CRH is a key modulator of parturition and cervical ripening its higher concentrations in the context of women with depression and anxiety could explain the increased risk of preterm birth seen in these women^26^,^27^. However, not all studies have shown an increase in CRH levels in women with depression^28^.

One possibility to explain the disparities in neonatal outcomes seen in our study is the “weathering hypothesis”^29^. In our study there were greater numbers of indigenous and Asian women as well as those born outside of Australia in the public cohort. Given that Australia accepts a large number of migrants and refugees from some of the most socially disadvantaged regions of the world, the psychosocial stress resulting from their cumulative experiences may prematurely “age or weather” the reproductive system, contributing to the increased rates of poorer outcomes seen in this study^29^,^30^. Although it may be speculative at this stage, it is possible that factors influencing perinatal outcomes may go beyond ethnicity, insurance status, maternal co-morbidities and age but may also include a woman’s previous social and psychological experiences.

Health professionals should be cognizant of the perinatal complications of mental health disorders both in pregnant women as well as women contemplating pregnancy so that appropriate surveillance, both maternal and fetal can be instituted. Women also need to be counselled about some of the potential intrapartum and perinatal complications associated with mental health problems and this is particularly important in the context of those on psychotropic medication. Although there isn’t robust evidence to suggest that preconception stabilisation of mental health improves perinatal outcomes, as a general principle this should be the aim. In addition, post conception professional and family support as well as optimisation of medication is critical in the management of these women.

The strengths of our study are the large sample size with prospectively collected EPDS scores at the booking visit and detailed perinatal outcomes. The limitations of our study include its retrospective nature, the lack of additional information on lifestyle, smoking, socioeconomic status, family support, substance abuse and use of non disclosed psychotropic or other medication, all of which could have influenced our results. Our results could also have been confounded if depression occurred after the initial EPDS screen. Nevertheless, our key findings particularly the increased risk of emergency caesarean section for fetal concerns and the adverse neonatal outcomes especially neonatal death are worrying and warrant further investigation.

## Additional Information

**How to cite this article**: Navaratne, P. *et al*. Impact of a high Edinburgh Postnatal Depression Scale score on obstetric and perinatal outcomes. *Sci. Rep.* **6**, 33544; doi: 10.1038/srep33544 (2016).

## Figures and Tables

**Table 1:** Maternal demographics.

	Whole cohort[n = 26110]	EPDS < 9[n = 20512]	EPDS ≥12[n = 2708]	p value
Age*	29 [26–33]	29 [26–33]	28 [24–33]	<0.001
BMI categories (kg/m^2^)				
Underweight (<18.5)	2131 (8.2)	1650 (8.0)	229 (8.5)	0.993
Normal (18.5–24.9)	14339 (55.0)	11385 (55.5)	1408 (52.0)	0.001
Overweight (25.0–29.9)	5336 (20.4)	4139 (20.2)	583 (21.5)	0.057
Obese1 (30.0–34.9)	2360 (9.0)	1842 (9.0)	251 (9.3)	0.645
Obese2 (35.0–39.9)	954 (3.7)	742 (3.6)	108 (4.0)	0.287
Obese3 (≥40.0)	666 (2.6)	510 (2.5)	92 (3.4)	0.003
Ethnicity				
Caucasian	16202 (62.1)	13066 (63.7)	1519 (56.1)	<0.001
ATSI	569 (2.2)	389 (1.9)	103 (3.8)	<0.001
Asian	4161 (16.0)	3254 (15.9)	367 (13.6)	0.132
Indian	764 (2.9)	605 (3.0)	61 (2.3)	0.081
Other	4368 (16.7)	3164 (15.4)	650 (24.0)	<0.001
Born outside Australia	11314 (43.3)	8724 (42.5)	1231 (45.5)	<0.001
Parity				
P0	12224 (46.8)	9557(46.6)	1274 (47.0)	0.099
P ≥ 1	13855 (53.1)	10926 (53.3)	1434 (53.0)	0.073
Hypertension including PET	1435 (5.5)	1098 (5.4)	176 (6.5)	0.043
Gestational Diabetes	1705 (6.5)	1308 (6.4)	188 (6.9)	0.668
Thyroid Disease	1094 (4.2)	827 (4.0)	131 (4.8)	0.013
Total length of labour (minutes)*	365 [216–570] n = 21388	366 [215–570] n = 16885	352 [216–561] n = 2181	0.252
Length of stay (days)*	2 [1–3] n = 26085	2 [1–3] n = 20496	2 [2,3] n = 2701	<0.001

EPDS: Edinburgh Postnatal Depression Scale.

BMI: Body Mass Index.

PET: Pre-eclampsia.

ATSI: Aboriginal and Torres Strait Islander.

Data presented as Number (percentage) unless otherwise specified.

^*^Data presented as Median [Interquartile range].

**Table 2:** Modes of Delivery.

	Whole cohort[n = 26110]	EPDS < 9[n = 20512]	EPDS ≥12[n = 2708]	p value
SVD	15182 (58.1)	11982 (58.4)	1562 (57.7)	0.082
Instrumental	3252 (12.5)	2591 (12.6)	303 (11.2)	0.101
Caesarean section	7674 (29.4)	5938 (29.0)	843 (31.1)	0.002
Planned CS	3107 (11.9)	2408 (11.7)	329 (12.1)	0.373
Emergency CS	4567 (17.5)	3530 (17.2)	514 (19.0)	0.006
Category 1	1161 (4.4)	892 (4.3)	142 (5.2)	0.024
Category 2	2320 (8.9)	1811 (8.8)	244 (9.0)	0.554
Category 3	835 (3.2)	638 (3.1)	100 (3.7)	0.071
Category 4	120 (0.46)	99 (0.48)	11 (0.41)	0.717

SVD: Spontaneous vaginal delivery.

CS: Caesarean section.

Data presented as Number (percentage) unless otherwise specified.

**Table 3:** Perinatal outcomes.

	Whole cohort [n = 26110]	EPDS < 9 [n = 20512]	EPDS ≥12 [n = 2708]	p value
Gestation at delivery*	39 [38–40]	39 [38–40]	39 [38–40]	<0.001
Gestation category				
Preterm (<37 weeks)	2376 (9.1)	1770 (8.6)	316 (11.7)	<0.001
Birth weight* (g)	3408 [3048–3758]n = 26109	3420 [3066–3765]n = 20512	3330 [2950–3696.25]n = 2707	<0.001
BW < 10th centile	2530 (9.7)	1881 (9.2)	340 (12.6)	<0.001
BW < 5th centile	1235 (4.7)	901 (4.4)	169 (6.2)	<0.001
Apgar < 7 at 5 min	561 (2.1)	412 (2.0)	84 (3.1)	<0.001
Umbilical arterial pH < 7.2/Lactate > 4	244 (0.93)	196 (0.95)	23 (0.85)	0.623
NICU admission	1019 (3.9)	763 (3.7)	126 (4.7)	0.511
Severe respiratory distress syndrome	1353 (5.2)	1055 (5.1)	152 (5.6)	0.351
Infection	517 (2.0)	405 (2.0)	60 (2.2)	0.366
Neonatal resuscitation	8110 (31.1)	6269 (30.6)	932 (34.4)	<0.001
Neonatal death	45 (0.17)	26 (0.13)	13 (0.48)	0.010

NICU: Neonatal Intensive Care Unit.

BW: Birth weight.

Data presented as Number (percentage) unless otherwise specified.

*Data presented as Median [Interquartile range].

NB: Some denominators vary because of the number of non-recorded data.
